# CD9 regulates proliferation, invasion, migration and radioresistance of esophageal squamous cell carcinoma by activating AKT/GSK3β signaling pathway

**DOI:** 10.3389/fonc.2025.1625120

**Published:** 2025-08-08

**Authors:** Liubing Hou, Zengyao Hao, Ge Zhang, Jiayuan Li, Yu Wang, Xiaoying Xue, Huandi Zhou

**Affiliations:** ^1^ Department of Radiotherapy, The Second Hospital of Hebei Medical University, Shijiazhuang, Hebei, China; ^2^ Department of Central Laboratory, The Second Hospital of Hebei Medical University, Shijiazhuang, Hebei, China; ^3^ Hebei Key Laboratory of Etiology Tracing and Individualized Diagnosis and Treatment for Digestive System Carcinoma, The Second Hospital of Hebei Medical University, Shijiazhuang, Hebei, China

**Keywords:** esophageal squamous cell carcinoma, radioresistance, prognostic biomarker, CD9, Akt/GSK3β pathway

## Abstract

**Background:**

Radioresistance poses a major therapeutic challenge in ESCC, significantly impacting patient prognosis. CD9, as a crucial membrane regulatory protein, exhibits dual regulatory roles in various cancers, yet its precise mechanism in ESCC radioresistance remains unclear. This study aims to systematically elucidate the molecular mechanisms by which CD9 regulates malignant phenotypes and radiosensitivity through the AKT/GSK3β signaling pathway in ESCC.

**Methods:**

TCGA data, ESCC tissues microarray, *in vitro* experiments, and patient cohorts were utilized to investigate the expression patterns, functional mechanisms, and clinical relevance of CD9 in ESCC. CD9 was overexpressed in Eca109 cells (with low baseline expression) and knocked down in TE13 cells (with high endogenous expression). Functional assays, including proliferation, migration, invasion, and radioresistance tests, were conducted. Western blotting was used to explore the changes in key molecules of the AKT/GSK3β pathway. Survival analysis was performed on 82 ESCC cases from TCGA. Retrospectively collected radical radiotherapy specimens (n=14) from our institutional biobank underwent immunohistochemical quantification of CD9 expression correlated with survival outcomes.

**Results:**

TCGA data analysis and 32 paired ESCC and adjacent non-tumorous tissues microarray revealed that CD9 expression was higher in ESCC tissues than in normal tissues, and was associated with tumor stage or lymph node metastasis. Functional validation demonstrated CD9 overexpression in Eca109 cells augmented proliferation, migration and invasion capacity, while enhancing radioresistance (SF2 increased from 0.488 to 0.596, SER decreasing to 0.888). Conversely, CD9 knockdown in TE13 cells reduced SF2 from 0.579 to 0.461, and SER up to 1.244. Mechanistically, CD9 modulated p-AKT (ser473) and p-Gsk3β (ser9) levels increased to 1.95-fold and 1.42-fold in overexpression models, respectively, with 58% and 33% reductions in knockdown group. TCGA cohort analysis (n=82) revealed no significant OS/DSS/PFI differences by CD9 expression *(P>0.05*). Intriguingly, radiotherapy subgroup analysis (n=29) suggested CD9-low patients exhibited a trend toward prolonged OS *(P=0.067*) DSS (*P=0.067*) and PFI (*P=0.179*). ROC demonstrated notable predictive capacity for 3-/5-year OS (AUC=0.681/0.851), DSS (AUC=0.651/0.778) and PFI (AUC=0.853/0.824).

**Conclusions:**

CD9 promotes ESCC progression and radioresistance by activating the AKT/GSK3β pathway and holds promise as a potential prognostic biomarker and therapeutic target for ESCC.

## 1 Introduction

Esophageal cancer (EC) is a highly aggressive form of cancer, and ranks as the sixth leading cause of cancer-related deaths globally (1). It primarily consists of two subtypes: squamous cell carcinoma (ESCC) and adenocarcinoma (EAC). Notably, ESCC represents about 90% of all EC cases, with the highest rates found in Asian and African countries, particularly in China (2). Radiation therapy plays a crucial role in the comprehensive treatment of cancer, especially as a curative approach for ESCC. However, a significant challenge in clinical practice is the inherent resistance of tumor cells to radiation (3). Despite advancements in precision radiotherapy that have enhanced local control rates, approximately 40-60% of patients still face local recurrence and distant metastasis, largely due to this radiation resistance (4). This clinical dilemma underscores the pressing need to uncover the molecular mechanisms underlying radioresistance, as well as to develop new predictive biomarkers and therapeutic targets.

Tetraspanin is a highly conserved transmembrane protein family with 33 unique members in humans, playing important roles in many different cellular environments (5). Members of this family are widely involved in various physiological and pathological processes, including fertilization, cell adhesion, cell movement, and tumor invasion (6, 7). Tetratransmembrane proteins are often used as molecular promoters or adaptor proteins in terms of function, forming a “tetratransmembrane protein network” or microdomains rich in tetratransmembrane proteins by constructing interaction networks between molecules on the cell surface (6, 8). It is worth noting that some transmembrane proteins play important roles in regulating various cellular processes such as adhesion, migration, fusion, and signal transduction within the tumor microenvironment, plays an essential role in cancer biology (9).

CD9, as an important member of the tetraspanin family, has gradually gained attention in cancer research in recent years and has become a hot research topic in the field of tumor biology. The molecular structure of CD9 includes four transmembrane domains, two outer rings, and one inner ring, which enables it to interact with various cell surface receptors and ligands, thus playing an important role in cell signaling (10). It has shown that CD9 plays a crucial role in biological behaviors such as cell proliferation, migration, and apoptosis, particularly in the metastasis and invasion of tumor cells (11). CD9 exhibits a double-edged sword effect in various types of tumors, serving as both a tumor suppressor and a promoter of cancer in tumor occurrence/metastasis (12). For example, in malignant mesothelioma, higher levels of CD9 expression are associated with a better prognosis, while reducing its expression leads to increased cell migration, suggesting that the lack of CD9 may contribute to greater invasiveness (13). In contrast, in pancreatic cancer, CD9 plays a role by interacting with α-secretases, such as ADAM9, ADAM10, and ADAM17, to influence the Notch signaling pathway, which in turn promotes cancer development (14). This functional heterogeneity underscores the tissue-specific roles of CD9 in tumor biology. In ESCC, CD9 expression has been associated with lymph node metastasis (15). However, its potential involvement in radioresistance has not been thoroughly investigated. This study seeks to explore the oncogenic role of CD9 in the progression of ESCC, focusing on its regulatory effects on radioresistance. Ultimately, the goal is to establish a theoretical basis for predicting sensitivity to radiotherapy and for the development of new radiosensitizers.

## 2 Materials and methods

### 2.1 Patient specimen collection

Collect biopsy specimens and clinical follow-up information from 14 esophageal squamous cell carcinoma patients (aged 51–84 years) admitted to the Radiotherapy Department of the Second Hospital of Hebei Medical University and undergoing radical radiotherapy. After inquiring about the medical history, clinical laboratory examination, and histopathological diagnosis, it was diagnosed as esophageal squamous cell carcinoma. The verbal consent was obtained and all selected patients in this experiment were approved by the Ethics Committee of the Second Hospital of Hebei Medical University (2023-R042).

ESCC tissue microarray containing 32 paired ESCC tissues and adjacent non-tumorous tissues were purchased from *Servicebio. Co., Ltd* (EC-2302, Wuhan, China).

### 2.2 Cell culture

The ESCC cell lines TE13 and Kyse30 used in this study were gifted to Professor Gao Xianshu’s laboratory at Peking University First Hospital, while Eca109 and Kyse150 were purchased from Shanghai Saibai Kang and Zhong Qiao Xin Zhou Biotechnology Co., Ltd, respectively. Place the cells in RPMI-1640 medium containing 10% fetal bovine serum, 100mg/mL streptomycin, and 100U/mL penicillin, and culture at 37°C in a 5% CO2 incubator.

### 2.3 RNA extraction and RT qPCR

According to the experimental group, plate the cells with a density of 80% to 90%, and transfect, adhered overnight. Prepare each component according to the instructions of lip3000 for the experiment, and change the medium after transfection for 4–6 hours. After 24 h, 5×10^5^cells/well were seeded in 6-well plates, and the medium with the optimal G418 (shRNACD9, expression vector: pGPU6/RFP/Neo) or Hygromycin (CD9, expression vector: pCMV3-C-OFPSpark) concentration was added the next day, replaced every 3–5 days and halving the G418 or Hygromycin concentration at day 10. resistant clones usually appeared after 14 days. These clones were marked, isolated using cloning rings or a cell scraper, and expanded stepwise in plates with 25% of the optimal G418 or Hygromycin concentration, and then, further amplification and cryopreservation.

Extract total RNA from cells using TRIZOL. Refer to the instructions of the reverse transcription kit (Yeasen, Shanghai, China) for reverse transcription, and synthesize complementary DNA (cDNA) with 2.0 ug of total RNA. Use RT qPCR kit (Yeasen, Shanghai, China) to detect the mRNA expression level of CD9. GAPDH serves as an internal reference gene. The specific primers used are showed in **Supplementary Table S1**. The RT qPCR amplification conditions are pre denaturation: 95 °C, 5 minutes → [Denaturation: 95 °C, 10s → Annealing/Extension: 60 °C, 30s] × 40 cycles. The relative expression levels of target genes were calculated using the 2^-ΔΔCt^ method, with the untreated group as the control and GAPDH as the internal reference. Here, ΔCt = Ct target gene - Ct GAPDH, and ΔΔCt = ΔCt experimental group - ΔCt control group.

### 2.4 Western blotting

Collecting the cells, and washing the cells with pre cooled PBS, RIPA lysis buffer containing protease inhibitors was added and lysed on ice for 15 minutes. Centrifuge at 12000g at 4 °C for 15 minutes. Measure the concentration using BCA protein quantification kit (Solarbio, Beijing). Protein samples were mixed with 5 × Loading Buffer, denatured in a metal bath at 100 °C for 15 minutes, and stored at -80 °C for future use. Take 20 μg sample from each well and add 10% sodium dodecyl sulfate polyacrylamide gel electrophoresis (SDS-PAGE) to carry out the experiment (3). Antibodies CD9 (Proteintech, 1:5000), GAPDH (Proteintech, 1:20000), AKT/p-AKT (CST, 1:1000/2000), GSK3β/p-GSK3β (Affinity Biosciences, 1:1000). After development with ECL chemiluminescence reagent (Biosharp, China), images were collected using ChemiDoc imaging system (Bio Rad) and analyzed using Image J software.

### 2.5 CCK8 and EdU detection of cell proliferation ability

CCK-8 method: After transfection, cells were seeded in a 96 well plate (3×10³/well), and cell viability was monitored daily using 10% CCK-8 reagent (MCE, USA) for 7 consecutive days. After incubation at 37 °C in the dark for 1 hour, the absorbance value at 450 nm was measured.

EdU method: After transfection, cells were seeded onto a 96 well plate (1×10^4^/well). The cells were incubated with EdU kit A solution (1000:1, Guangzhou Ruibo Co., Ltd.) at 37 °C for 1 hour, washed, fixed in methanol for 30 minutes, punched, sealed, and stained. Finally, take photos under a fluorescence microscope and count the proportion of positive cells.

### 2.6 Transwell invasion assay detects cell invasion and migration ability

Migration experiment: Add 2 × 10^4^ cell suspension (200 μL serum-free medium) to Transwell upper chamber (8 μm pore size, Corning^®^ company), and add 600 μL of 10% FBS medium to the lower chamber. After 24 hours, fix with methanol, stain with 0.1% crystal violet, and randomly count migrating cells in 5 fields. Invasion experiment: Matrigel matrix gel (BD Biosciences) was pre encapsulated in Transwell chambers, and the remaining steps were the same as the migration experiment.

### 2.7 Cell scratch assay to detect cell migration ability

Inoculate logarithmic growth phase cells onto a 6-well plate and culture for 24 hours. Use a 10uL pipette tip to draw an “L” - shaped scratch on the bottom of the culture plate, culture in serum-free medium, observe under a microscope, and take photos of the fixed location at 0/12/24/48 hours for recording. Use Image J software to analyze data and calculate cell migration rate.

### 2.8 Cell irradiation and colony formation assay

When the cell density reaches 70%~90%, the Eleketa synergy linear accelerator 6MV X-ray is used to irradiate cells at a dose rate of 200cGy/min, with doses of 0, 2, 4, 6, 8, or 10 Gy, respectively. Inoculate in 6-well plates according to gradient density and culture for 10–14 days. After methanol fixation and crystal violet staining, the number of clones of>50 cells were counted. A single click multi-target model was used to fit the survival curve, and multiple radiobiological parameters such as D0 (mean lethal dose), Dq (quasi-threshold dose), N (extrapolation number), SF2 (surviving fraction at 2 Gy), and SER (sensitization enhancement ratio) were calculated.

### 2.9 Immunohistochemistry

After baking at 65°C for 4 hours, ESCC tissue sections were subjected to gradient dewaxing (xylene: 100% → 85% → 75%, 10 min/step) and ethanol gradient hydration (100%→25%, 8 min/step) in sequence. Antigen repair was performed using 1 × EDTA buffer (95°C water bath for 1 hour) and 3% H_2_O_2_ blocking endogenous peroxidase (30 minutes, room temperature). Incubate CD9 primary antibody (1:1000, overnight at 4°C) and HRP labeled secondary antibody (1h at 37°C) in sequence, and perform DAB staining (1min, under microscopic monitoring). After counterstaining with hematoxylin, the gradient dehydration was transparent, and neutral gum was used to seal the slices. Image J analysis showed staining intensity.

### 2.10 Kyoto encyclopedia of genes and genomes pathway analysis

The mRNA expression profiles and the corresponding clinical information from ESCC patients (n=82) and normal (n=11) were downloaded from TCGA dataset (https://portal.gdc.cancer.gov/). Using Sangerbox online tool (16) (http://sangerbox.com/home.html), ID conversion (EntrezID)→ Genesymbol), standardization [log2 (X+1)]. Then, the R software package limma (version 3.40.6) was used for differential analysis to obtain differentially expressed genes between different comparison groups and control groups. For gene set functional enrichment analysis, we use the KEGG rest API(https://www.kegg.jp/kegg/rest/keggapi.html) to obtain the latest KEGG pathway gene annotations as background. We used the R software package clusterProfiler for enrichment analysis to obtain the results of gene set enrichment. Set the minimum gene set to 5 and the maximum gene set to 5000, with a P <0.05 and an FDR <0.05, which were considered statistically significant.

### 2.11 Statistical analysis

All experiments were repeated at least three times. All data were presented as means ± SD. All statistical analyses were conducted using GraphPad Prism 8.0 software. paired or unpaired Student’s *t*-test or One/*Two-way ANOVA* was applied. The statistically significant p values were labeled as follows: *P < 0.05, **P < 0.01, ***P < 0.001.

## 3 Results

### 3.1 CD9 expression in esophageal cancer based on TCGA

**Figure 1:**
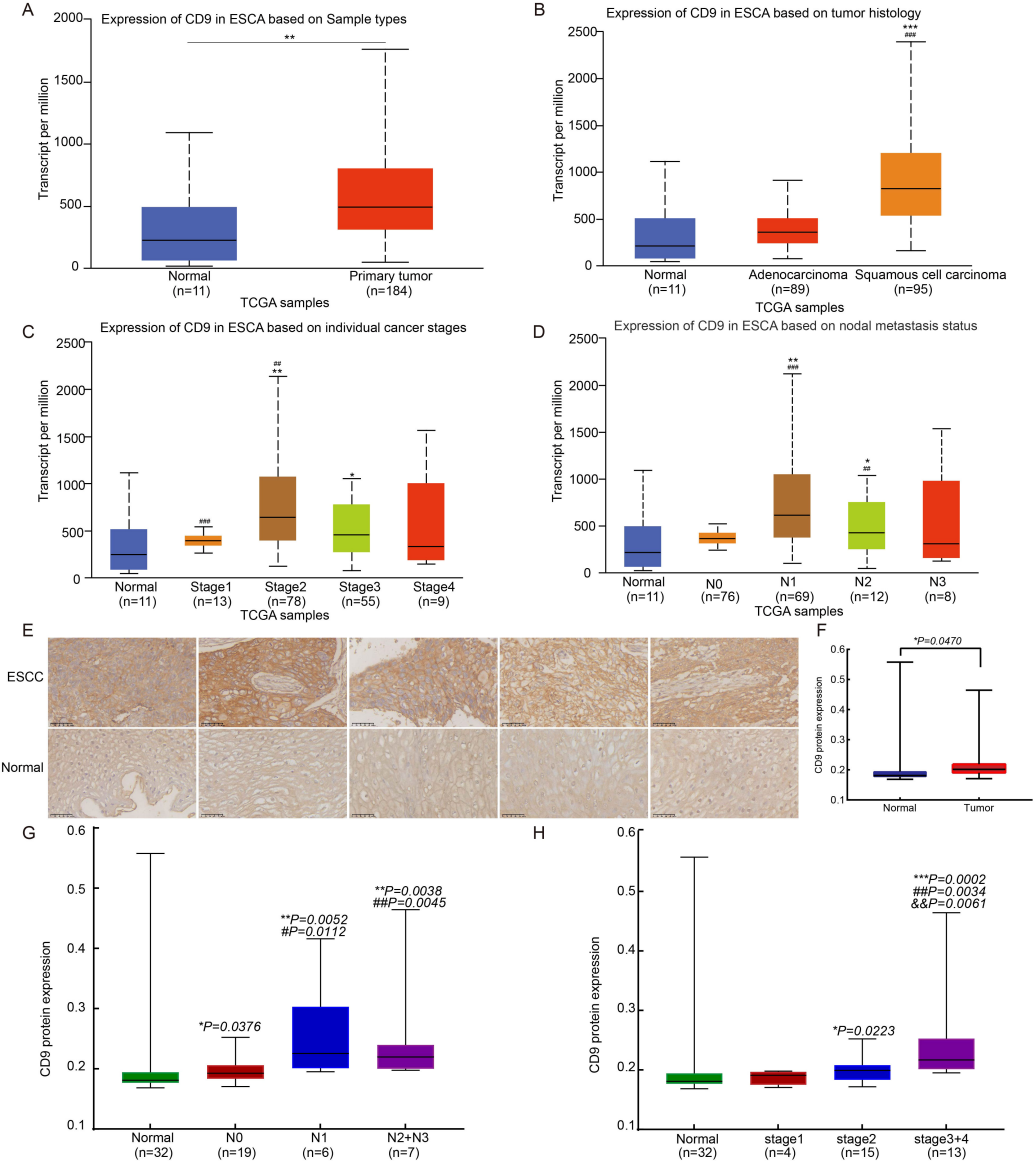
The expression of CD9 in ESCC. **(A)** The comparison of CD9 mRNA expression between normal controls and ESCA in TCGA database (**vs Normal, *P<0.01*). **(B)** The comparison of CD9 mRNA expression in esophageal normal control, EA, and ESCC in TCGA database (***vs Normal, *P<0.001*; ### vs adenocarcinoma, *P<0.001*). **(C)** The comparison of CD9 mRNA expression between normal controls and patients with different stages of ESCA in TCGA database (*vs Normal, #vs Stage I; * *P<0.05*, ##/**P<0.01, ###*P<0.001*). **(D)** The comparison of CD9 mRNA expression between esophageal normal controls and ESCA patients with different lymph node metastases in TCGA database (*vs Normal, #vs N0; *P<0.05, **P<0.01, ###*P<0.001*). **(E)** The CD9 expression in ESCC and paracancerous from 33 ESCC patients detected by IHC. (400×). **(F)** The difference of CD9 expression in ESCC and paracancerous from 32 ESCC patients. (*vs Normal, * *P<0.05*). **(G)** The comparison of CD9 protein expression between normal controls and patients with different stages of ESCC in ESCC tissue microarray (*vs Normal, #vs Stage I; */#*P<0.05*, ##/**P<0.01). **(H)** The comparison of CD9 protein expression between esophageal normal controls and ESCC patients with different lymph node metastases in ESCC tissue microarray (*vs Normal, #vs N0, &vs N2; * *P<0.05*, ##/&&*P<0.01*, ***P<0.001).

Analysis of TCGA data via UALCAN revealed significantly elevated CD9 mRNA expression in esophageal cancer tissues (n=184) compared to normal esophageal samples (n=11) (*P=0.0082*, **Figure 1A**). Further stratified analysis by histological subtypes demonstrated that CD9 mRNA levels were markedly higher in esophageal squamous cell carcinoma (ESCC) than in esophageal adenocarcinoma (EAC) (*P<0.0001*, **Figure 1B**), suggesting a potential subtype-specific role of CD9 in ESCC pathogenesis. In addition, CD9 expression exhibited differences in different cancer stages (**Figure 1C**) and was significantly correlated with lymph node metastasis risk (**Figure 1D**). In order to further determine the role of CD9 in ESCC, IHC was used to detect the expression of CD9 in 32 pairs of ESCC and paired adjacent tissues. The results showed that compared with 32 adjacent tissues, CD9 expression was higher in esophageal cancer tissues (*P=0.047*) (**Figures 1E, F**). Furthermore, the correlation between CD9 and cancer stages or lymph node metastasis also were confirmed (as Stage 4 and N3 only have 2 individuals, Stage 4 and Stage 3 were merged for settlement, and N3 and N2 were merged for calculation) (**Figures 1G, H**). These findings imply that CD9 may promote ESCC progression by enhancing metastatic potential.

### 3.2 CD9 expression and intervention in ESCC cell lines

**Figure 2:**
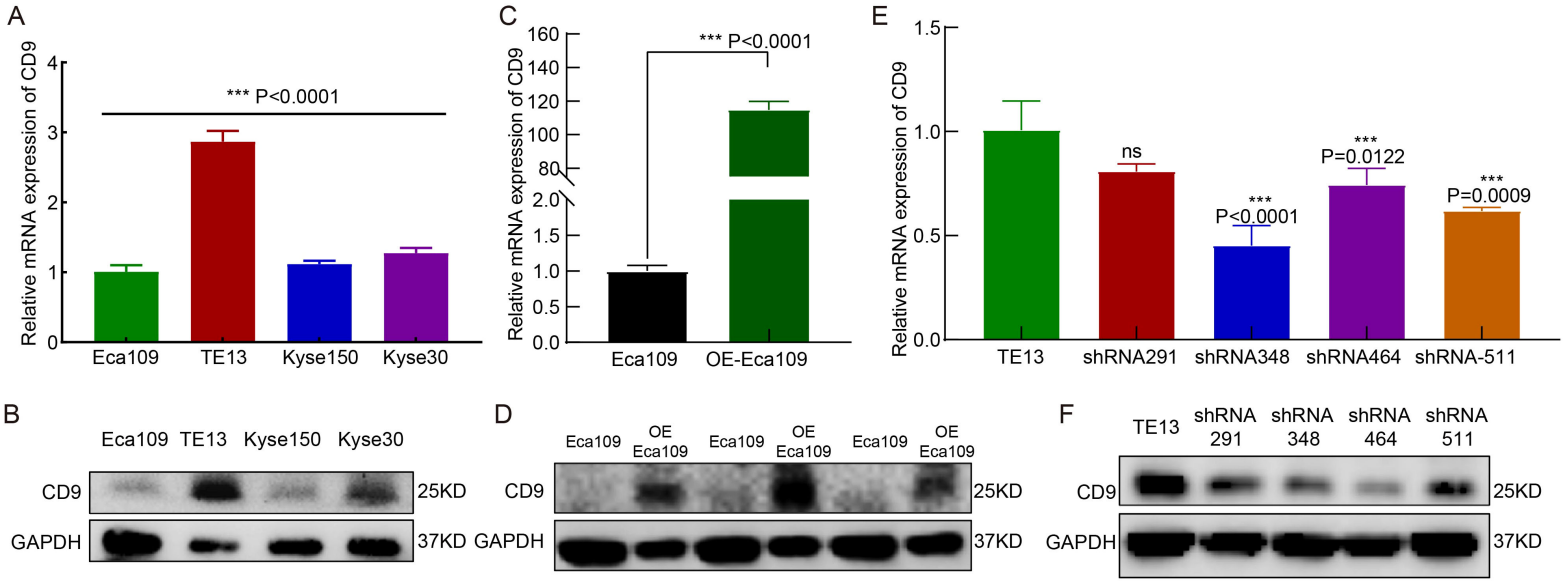
The expression and knockdown/overexpression validation of CD9 in ESCC cell lines. **(A, B)** The CD9 mRNA **(A)** and protein **(B)** expression in 4 ESCC cell lines detected by RT-qPCR and Western Blot, ***P<0.001. **(C, D)** The CD9 mRNA **(C)** and protein **(D)** expression changes in Eca109 cells after overexpression of CD9 detected by RT-qPCR and Western Blot, ***P<0.001. **(E, F)** The CD9 mRNA **(E)** and protein **(F)** expression changes in TE13 cells after knockdown of CD9 detected by RT-qPCR and Western Blot, ***P<0.001, ns, not significant.

RT-qPCR and Western blot analyses revealed heterogeneous CD9 expression across four ESCC cell lines (Eca109, TE13, Kyse150, and Kyse30). TE13 exhibited the highest CD9 mRNA and protein levels, whereas Eca109 showed the lowest expression (**Figures 2A, B**). Based on this baseline profiling, Eca109 and TE13 were selected for CD9 gain-of-function (overexpression) and loss-of-function (knockdown) experiments, respectively.

In Eca109 cells transfected with a CD9 overexpression plasmid (OE-Eca109), both mRNA and protein levels of CD9 were significantly elevated compared to vector controls (*P<0.0001*, **Figures 2C, D**). For TE13 knockdown, three independent shRNA constructs targeting CD9 (shRNA-CD9-homo-348, -464, and -511) were validated. All constructs effectively reduced CD9 mRNA and protein expression, with shRNA-CD9-homo-511 (shRNA-511) demonstrating the highest knockdown efficiency (**Figures 2E, F**). Consequently, shRNA-511 was selected for subsequent functional analyses.

### 3.3 CD9 overexpression promotes proliferation in ESCC cells

**Figure 3:**
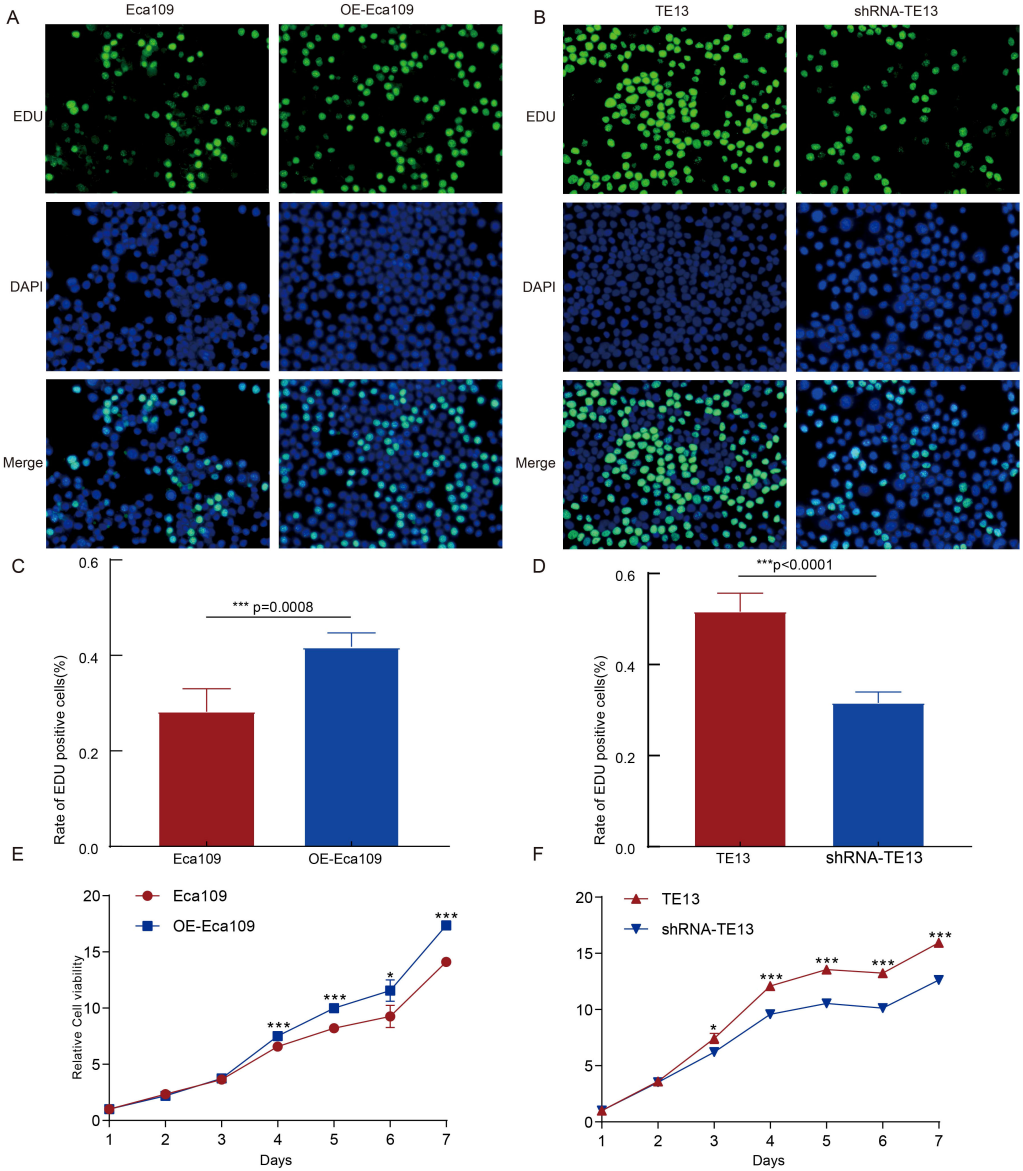
The effect of CD9 on the proliferation ability of ESCC cells. **(A, C)** The cell proliferation ability of Eca109 cells detected by EDU (***, P<0.001); **(B, D)** The cell proliferation ability of TE13 cells detected by EDU(***, P<0.001); **(E)** The cell proliferation ability of Eca109 cells detected by CCK-8 (*P<0.05; ***P<0.001); **(F)** The cell proliferation ability of TE13 cells detected by CCK-8 (*P<0.05, ***P<0.001).

To investigate the role of CD9 in ESCC cell proliferation, EdU and CCK-8 assays were performed. Experimental groups included: i) Eca109 cells (vector-transfected controls) vs. OE-Eca109 (CD9-overexpressing group), and ii) TE13 cells (scramble shRNA controls) vs. shRNA-TE13 (CD9-knockdown group). EdU analysis revealed a significant increase in the EdU-positive cell ratio in OE-Eca109 compared to controls (*P=0.0008*, **Figures 3A, C**). Conversely, CD9 knockdown in TE13 cells (shRNA-TE13) markedly reduced the EdU-positive cell ratio relative to scramble controls (*P<0.0001*, **Figures 3B, D**). Consistent with these findings, CCK-8 assays demonstrated enhanced cell viability in OE-Eca109 (**Figure 3E**) and attenuated viability in shRNA-TE13 (**Figure 3F**) over time. It collectively demonstrates that CD9 overexpression drives ESCC proliferation.

### 3.4 CD9 overexpression enhances migration and invasion in ESCC cells

**Figure 4:**
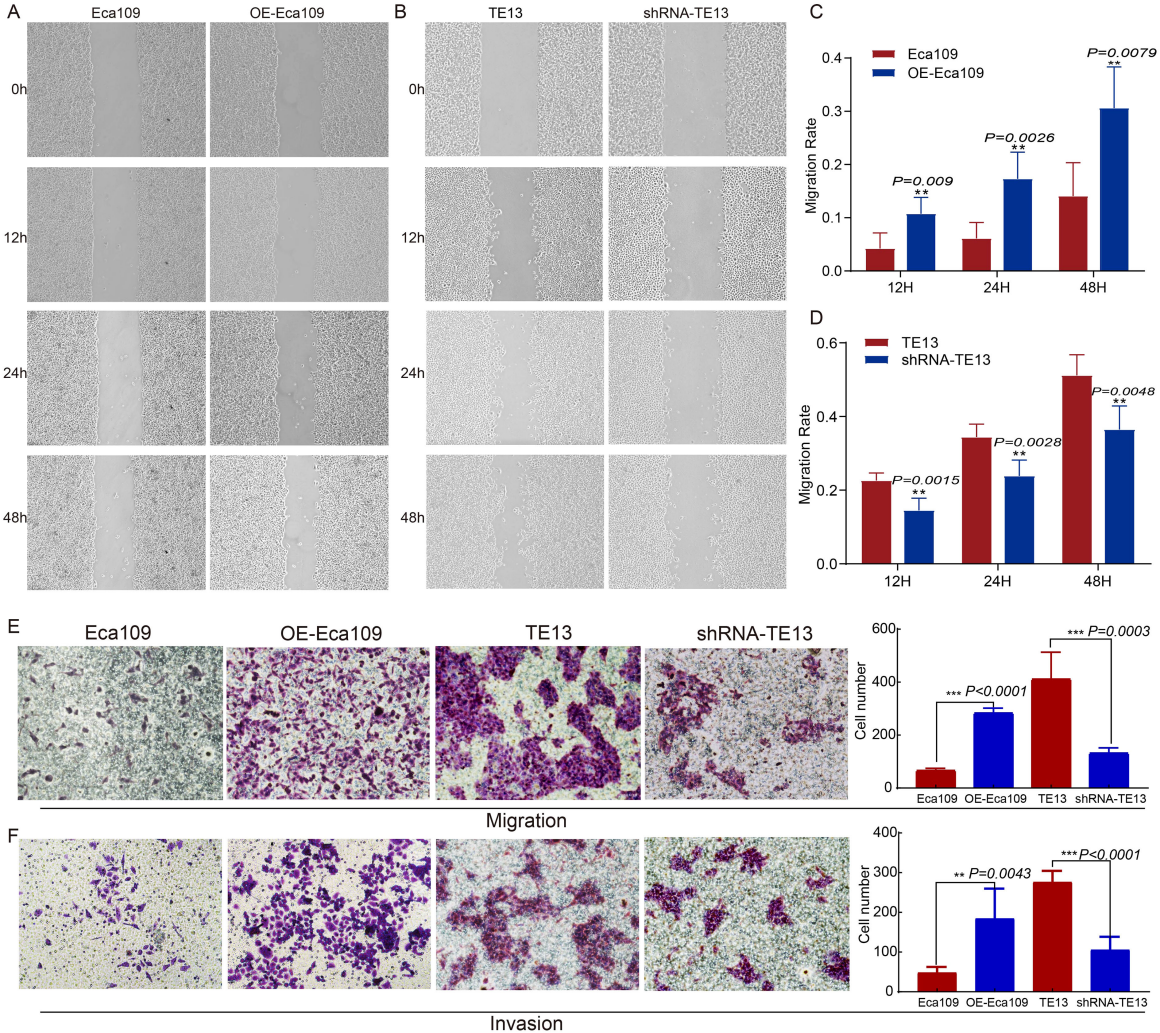
The effect of CD9 on the migration and invasion ability of ESCC cells (200×). **(A, C)** The changes in migration rate of Eca109 cells after overexpressing CD9 detected by scratch assay; **(B, D)** The changes in migration rate of TE13 cells after knocking down CD9 detected by scratch assay; **(E)** The changes in migration ability of ESCC cells after overexpressing or knocking down CD9 detected by transwell experiments. **(F)** The changes in invasion ability of ESCC cells after overexpressing or knocking down CD9 detected by transwell experiments. **P<0.01; ***P<0.001.

To investigate the role of CD9 in ESCC migration and invasion, wound-healing, transwell migration, and Matrigel invasion assays were conducted. CD9-overexpressing Eca109 (OE-Eca109) cells showed significantly accelerated scratch closure rates at 12 h, 24 h, and 48 h (*P<0.01* vs. control), whereas CD9-knockdown TE13 cells exhibited impaired closure (*P<0.01*) (**Figures 4A–D**). Transwell assays revealed that CD9 overexpression increased migratory cell counts (*P<0.0001*), while knockdown reduced them (*P=0.0003*) (**Figure 4E**). Similarly, Matrigel invasion assays demonstrated enhanced invasiveness in OE-Eca109 cells (*P=0.0043*) and suppressed invasiveness in shRNA-TE13 (*P<0.0001*) (**Figure 4F**). These findings suggest that CD9 acts as a pro-metastatic driver in ESCC.

### 3.5 CD9 overexpression confers radioresistance in ESCC cells

To investigate the role of CD9 in radioresistance, colony formation assays were performed on OE-Eca109 and shRNA-TE13 cells following irradiation (0,2,4,6,8,10 Gy). Radiobiological parameters, including D0, Dq, N, SF2, and SER, were calculated using the single-hit multi-target model.

The OE-Eca109 group exhibited significantly more colonies than the control under irradiation, whereas the shRNA-TE13 group showed a marked reduction in colony formation. Radiobiological parameter analysis revealed that CD9 overexpression elevated the D0 value from 1.202 Gy to 1.354 Gy and increased the Dq value from 1.388 Gy to 1.696 Gy, indicating enhanced sublethal damage repair capacity. Correspondingly, SF2 increased from 0.488 to 0.595. Conversely, CD9 knockdown in TE13 cells decreased the D0 value from 1.721 Gy to 1.383 Gy and reduced the Dq value from 1.472 Gy to 1.152 Gy, with SF2 declining from 0.579 to 0.461.

**Figure 5:**
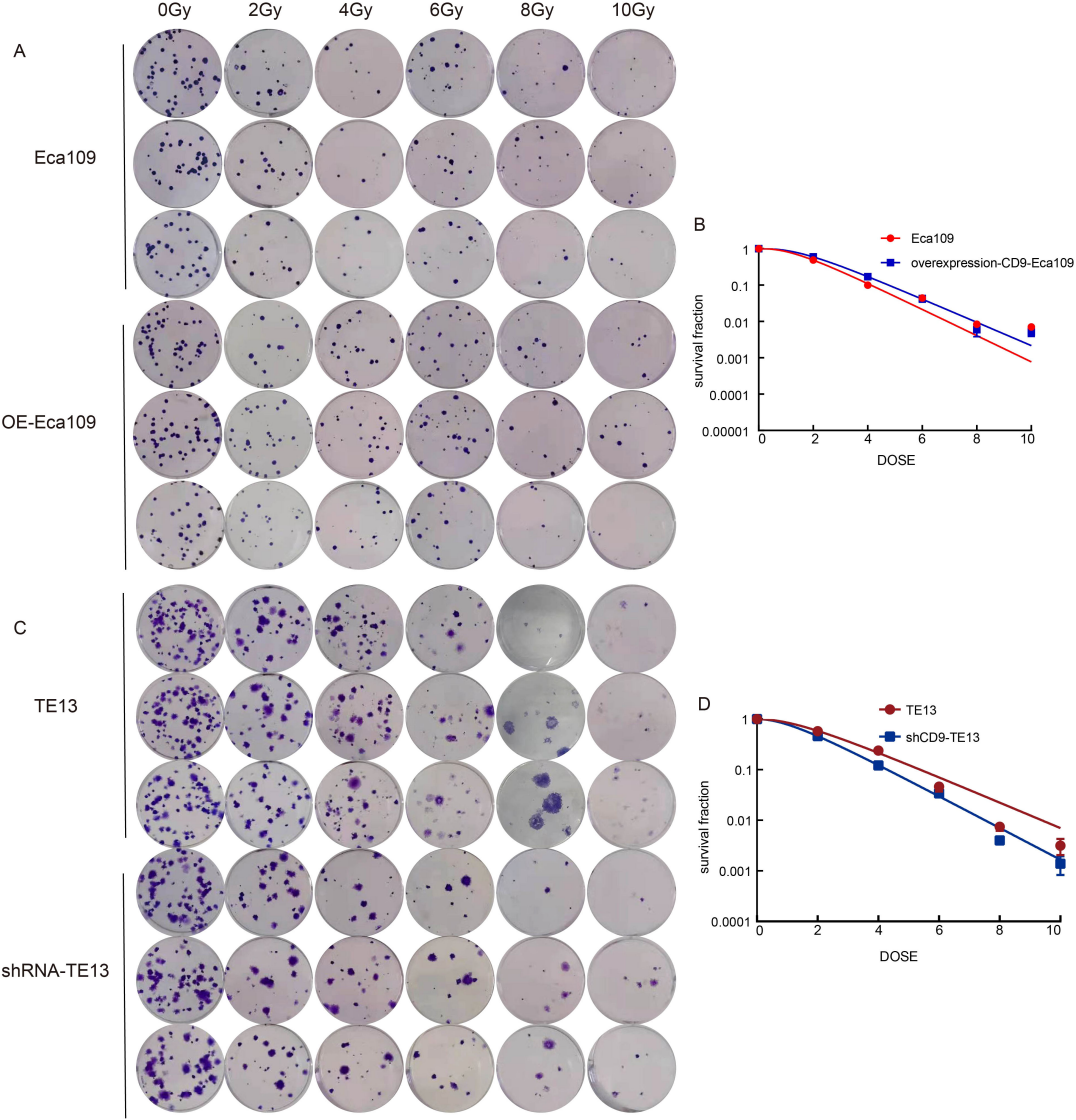
The relationship between CD9 and radiation resistance in ESCC. **(A, B)** The changes in radiation resistance of Eca109 cells with overexpressing CD9 through cloning formation experiments. **(C, D)** The changes in radiation resistance of TE13 cells with knocking down CD9 through cloning formation experiments.

SER analysis demonstrated that CD9 overexpression reduced the SER of Eca109 cells to 0.888, confirming enhanced radioresistance. In contrast, CD9 knockdown increased the SER of TE13 cells to 1.244, reflecting a significant radiosensitizing effect. These results collectively suggest that CD9 overexpression promotes radioresistance in ESCC cells by augmenting DNA repair efficiency and survival under irradiation (**Figure 5**, **Table 1**).

**Table 1:** The radiobiological parameters of click on multi-target model.

Cells	D0	Dq	N	SF2	SER
Eca109	1.202	1.388	3.174	0.488	0.888
OE-Eca109	1.354	1.696	3.499	0.596	
TE13	1.721	1.472	2.353	0.579	1.244
shRNA-TE13	1.383	1.152	2.300	0.461	

### 3.6 CD9 regulates the AKT/GSK3β signaling pathway in ESCC cells

**Figure 6:**
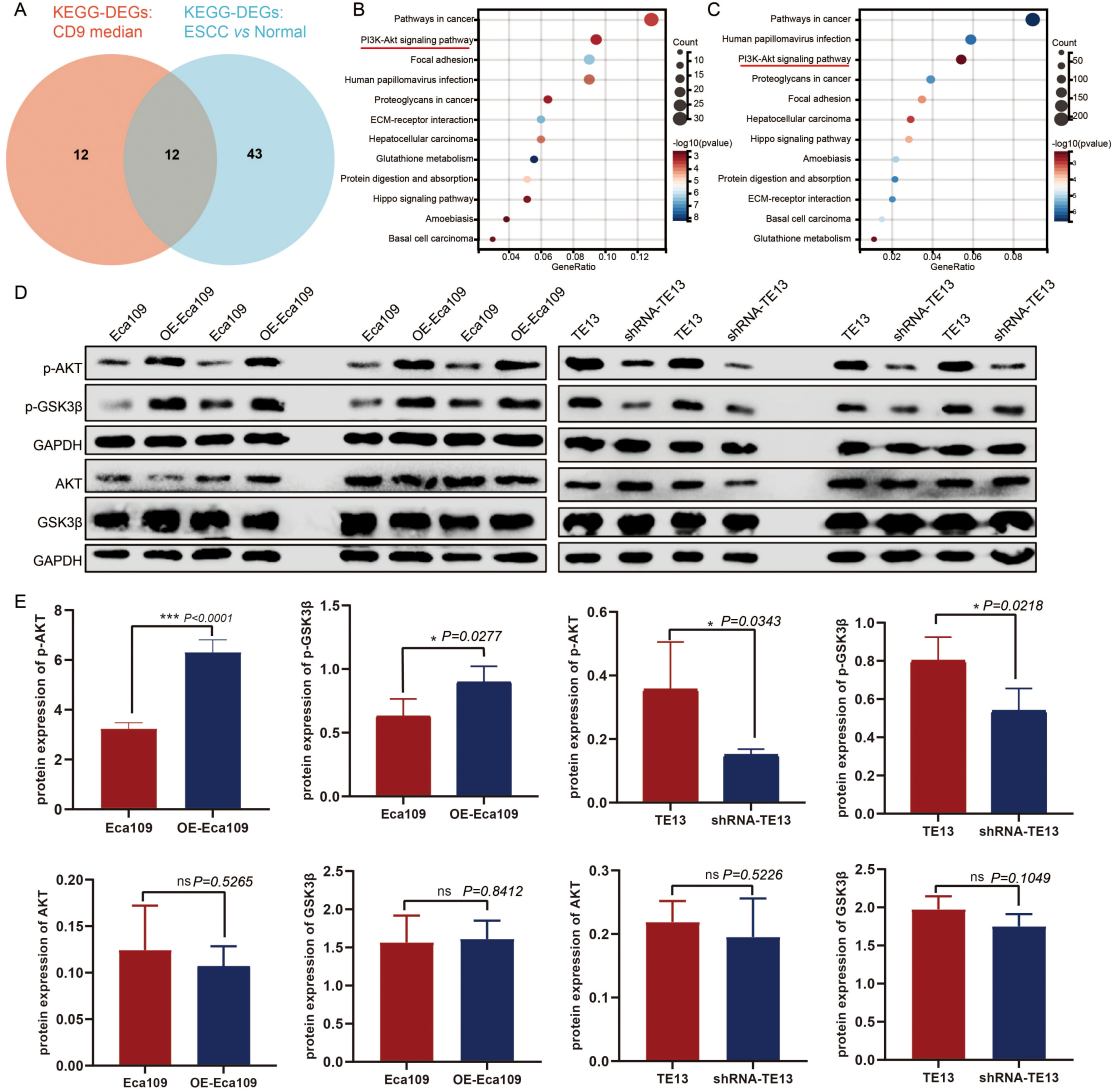
The regulatory effect of CD9 on the AKT/GSK3β signaling pathway in ESCC. **(A)** The Venn of KEGG-DEGs: CD9 median and KEGG-DEGs: ESCC vs normal. **(B)** The KEGG signaling pathways of DEGs in CD9 high expression group and CD9 low expression group. **(C)** The KEGG signaling pathways of DEGs in ESCC group and normal group. **(D)** Western blot detecting CD9 on the AKT/GSK3β signaling pathway in Eca109 and TE13 cells. **(E)** The Statistical results of CD9 on the AKT/GSK3β signaling pathway in Eca109 and TE13 cells detected by western blot, *P<0.05, ***P<0.001, ns: not significant.

To elucidate the mechanism by which CD9 promotes the malignant progression of ESCC, bioinformatics analysis based on TCGA-ESCC data were done. We conducted KEGG pathway analysis on differentially expressed genes (DEGs) between ESCC and adjacent normal tissues (|FC|>1.5, *P<0.05*) with screening criteria of *P<0.05* and *FDR<0.05*. Furthermore, ESCC samples were divided into high/low CD9 expression groups based on the median CD9 expression, and DEGs between these groups (|FC|>1.5, *P<0.05*) were also analyzed by KEGG (with same screening criteria). The intersection of the two analyses identified 12 common pathways, among which the PI3K/AKT signaling pathway ranked within the top three in both analyses (**Figures 6A–C**). Then, the protein expression levels of key components in the AKT/GSK3β signaling pathway were analyzed via Western blot in Eca109 (control and OE-Eca109) and TE13 (control and shRNA-TE13) cells. The results demonstrated that CD9 modulated p-AKT (ser473) and p-Gsk3β (ser9) levels increased to 1.95-fold (*P<0.0001*) and 1.42-fold (*P=0.0277*) in overexpression models, respectively, with 58% (*P=0.0343*) and 33%(*P=0.0218*) reductions in knockdown group. Notably, the total protein levels of AKT and GSK3β remained unchanged across all groups, indicating that CD9 specifically modulates the activation status rather than the abundance of these kinases (**Figures 6D, E**).

### 3.7 The relationship between CD9 expression and prognosis of ESCC patients

**Figure 7:**
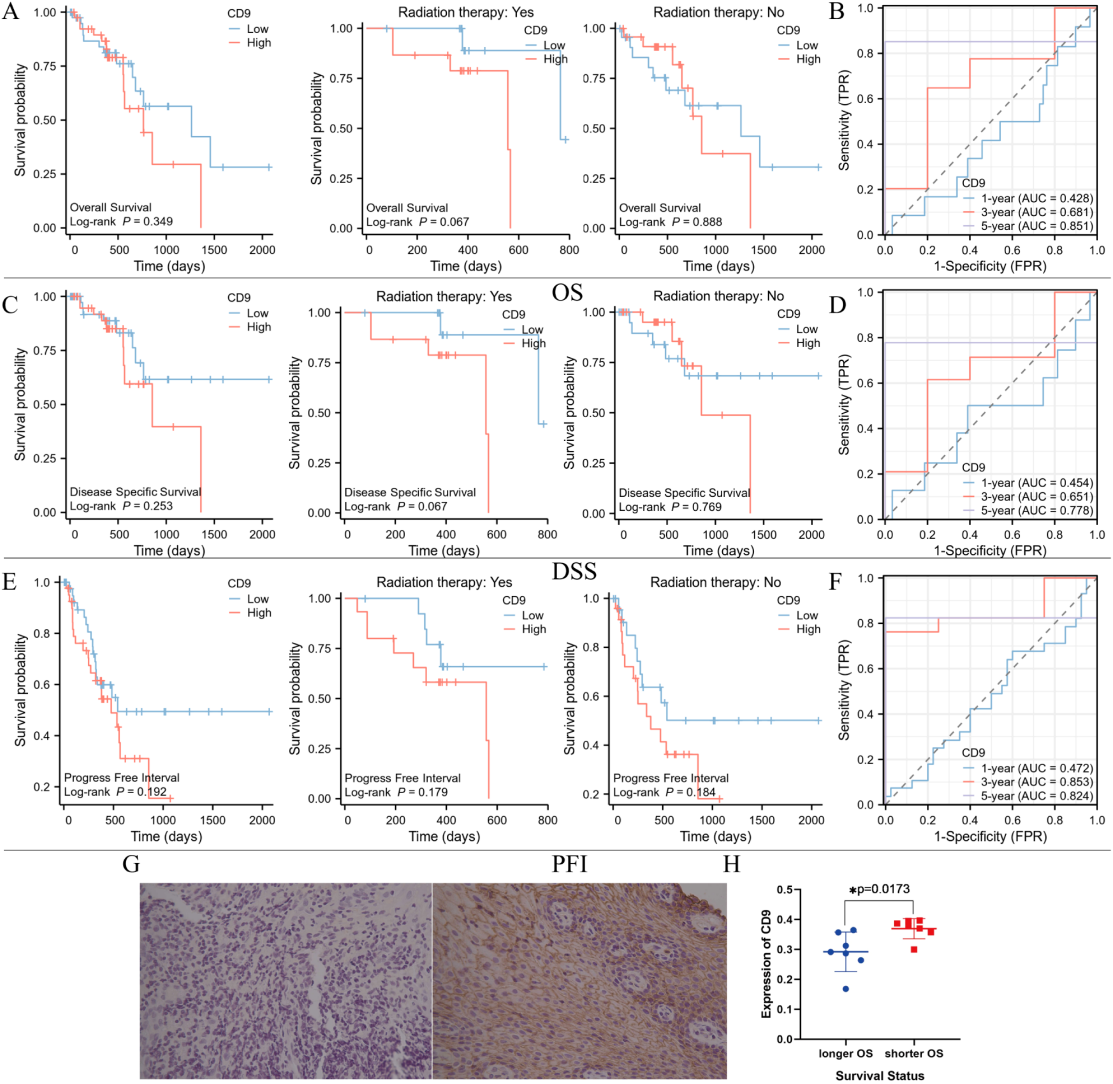
The relationship between CD9 expression level and prognosis of ESCC patients in TCGA database and our center. **(A)** Comparing the OS between high and low CD9 groups of ESCC patients in the TCGA database. **(B)** Time-dependent ROC analysis of CD9 based on patients’ OS in TCGA database. **(C)** Comparing the DSS between high and low CD9 groups of ESCC patients in the TCGA database. **(D)** Time-dependent ROC analysis of CD9 based on patients’ DSS in TCGA database. **(E)** Comparing the PFI between high and low CD9 groups of ESCC patients in the TCGA database. **(F)** Time-dependent ROC analysis of CD9 based on patients’ PFI in TCGA database. **(G)** The expression of CD9 in ESCC patients with longer (left) and shorter OS (right)(200×). **(H)** The differences in CD9 expression in ESCC patients with different OS (*P<0.05).

To investigate the relationship between CD9 and the prognosis of ESCC patients, Xiantao academic online tools was used. 82 patients with survival information were screened from the TCGA database, including 29 who received radiotherapy, 47 who did not receive radiotherapy, and 6 whose radiotherapy status was unknown. Divide patients into high and low CD9 expression groups based on the median CD9 expression value. Survival curve analysis showed that there were no significant differences in overall survival (OS), disease-specific survival (DSS), and progression free interval (PFI) between the CD9 high and low expression groups in ESCC patients and non-radiotherapy patients; However, among ESCC patients receiving radiotherapy, the OS (*P=0.067*), DSS (*P=0.067*) and PFI (*P=0.179*) of the CD9 low expression group showed a beneficial trend. Low expression of CD9 may increase the efficacy of radiotherapy in ESCC patients. Time dependent ROC analysis shows that the area under the ROC curve (AUC) based on OS is 0.428, 0.681, and 0.851 at 1 year, 3 years, and 5 years, respectively; The AUC based on DSS was 0.454, 0.651, and 0.778 at 1, 3, and 5 years, respectively; The AUC based on PFI was 0.472, 0.853, and 0.824 at 1, 3, and 5 years, respectively, indicating that CD9 has good predictive value for OS, DSS and PFI in ESCC patients at 3 and 5 years (**Figures 7A–F**).

To further evaluate the relationship between CD9 and the efficacy of radiotherapy for ESCC, biopsy specimens were collected from 14 ESCC patients who received radical radiotherapy at our center. Based on the median survival time (21 months), they were divided into a poor prognosis group and a good prognosis group. Immunohistochemistry was used to detect the expression of CD9, and the results showed that CD9 was mainly expressed on the cell membrane. Furthermore, analysis showed that compared with patients with shorter OS time, patients with longer OS time had lower CD9 expression levels (*P=0.0173*). These results suggest that CD9 may be a potential prognostic marker for radiotherapy efficacy in ESCC (**Figures 7G, H**).

## 4 Discussion

CD9, as an important member of the tetraspanin family, is a 24–27 kDa membrane protein (17, 18). Its role varies significantly depending on the type of cell and the partners it interacts with, participating in a wide range of cellular processes. These include facilitating cell-cell contact, mediating interactions between cells and the extracellular matrix, enabling integrin-dependent cell migration, and playing a part in signal transduction, membrane fusion, apoptosis, inflammation, proliferation, and differentiation (11, 19, 20). Interestingly, CD9 has a dual role in different types of tumors, acting both as a tumor suppressor and as a promoter of cancer during tumor development and metastasis.

Initially, CD9 was primarily recognized as a tumor suppressor. Research across various cancers, including prostate cancer (21–23), lung cancer (24, 25), colon cancer (26), breast cancer (27, 28) and mesothelioma (13), has demonstrated that CD9 can inhibit tumor growth and progression. This inhibition occurs mainly by restricting the movement of cancer cells and enhancing their adhesion to surrounding cells and the extracellular matrix. During the progression of prostate cancer, CD9 expression is significantly reduced or even lost due to the loss or mutation of its transcript, indicating that CD9 inactivation plays an important role in the disease’s progression (21) Notably, the expression of CD9 protein is lower in prostate cancer compared to benign prostatic hyperplasia, and this downregulation is associated with a shorter progression-free survival (PFS) in patients with high-grade prostate cancer. Consequently, CD9 has the potential to serve as a routine immunohistochemical biomarker for diagnosing and stratifying the risk of prostate cancer (23). Researchers have found that knocking out transmembrane tetraprotein CD9 in the Transgenic Adenocarcinoma of Mouse Prostate (TRAMP) model of newly diagnosed prostate cancer increases spontaneous metastasis in an organ specific manner (i.e., increases liver metastasis rather than lung metastasis) (22). CD9 is selectively absent in most small cell lung cancer cell lines and tissues, further leading to a highly malignant phenotype of small cell lung cancer. CD9 promotes apoptosis in small cell lung cancer cells by positively regulating the expression of calmodulin (25). In addition, in an *in-situ* lung tumor model established by lewis lung cancer (LLC) cells, both pre implantation of LLC cells transduced with CD9 adenovirus into the lungs and intratracheal administration of adenovirus encoding CD9 to tumor bearing mice reduced metastasis to mediastinal lymph nodes, suggesting that CD9 can prevent lymph node metastasis in primary lung cancer (24). CD9 can inhibit the proliferation and tumorigenicity of human colon cancer cells in colorectal cancer (26). The expression of CD9 in breast cancer is related to the epithelial phenotype and good prognosis of its patients. Knockout of CD9 in breast cancer cells can increase cell motility, showing an anti-tumor effect. However, in recent years, CD9 has also been found to be involved in tumor progression. Some clinical and experimental studies have shown that CD9 can also lead to increased proliferation, migration, and survival of cancer cells in various tissue types. The expression of CD9 is higher in primary and metastatic gastric cancer tissues than in adjacent tissues of the same patient, and high expression of CD9 is associated with vascular invasion, lymph node metastasis, and advanced stage (29). It was found that overexpression of CD9 can promote adhesion, migration and invasiveness in breast cancer cells (30). In addition, the level of CD9 in tumor and matrix immune cells of patients with invasive breast cancer has different roles. CD9 on matrix immune cells is related to longer disease-free survival, while CD9 on tumor cells is related to lymph nodes and distant metastasis (31). From this, it can be seen that CD9 has complex functional regulatory roles in different tissue types, once again confirming the tissue-specific characteristics of tetraspanin protein function.

At present, there is limited research on the role of CD9 in esophageal cancer. Imamura et al. conducted immunohistochemical analysis of CD9 expression in 108 cases of esophageal squamous cell carcinoma and found that its decreased expression was significantly correlated with tumor depth. They also found that the decrease in CD9 expression may promote lymph node metastasis in esophageal squamous cell carcinoma (32). Ye Tian et al. found that CD9 is highly expressed in ESCC tissues compared to normal esophageal tissues and tumor adjacent tissues. The expression of CD9 is associated with ESCC staging and lymph node metastasis, and the expression of CD9 in metastatic ESCC tissues is significantly higher than that in non-metastatic tissues, indicating that CD9 is a characteristic of malignant progression (15). In addition, based on single-cell analysis of differentially expressed genes in ESCC cells before and after neoadjuvant therapy, Chen et al. identified 12 key cisplatin sensitivity differentially expressed genes, including CD9, suggesting a correlation between CD9 and the efficacy of cisplatin neoadjuvant therapy in esophageal cancer. Therefore, the role of CD9 in esophageal squamous cell carcinoma is inconsistent and lacks systematic research, especially the role of CD9 in ESCC radiation resistance.

This study systematically revealed for the first time the expression characteristics, functional effects, and regulatory role of CD9 on radiosensitivity in ESCC. By combining bioinformatics analysis with functional validation, we found that CD9 is significantly upregulated in ESCC and is associated with tumor staging and lymph node metastasis, suggesting its possible involvement in tumor progression. Further *in vitro* experimental results showed that overexpression of CD9 can significantly promote the proliferation, migration, and invasion ability of ESCC cells, while enhancing their resistance to radiotherapy. Radiobiological parameter analysis showed that high expression of CD9 can enhance SF2 and reduce SER, suggesting that it may participate in the mechanism of radiation resistance by enhancing DNA damage repair ability.

Based on bioinformatics analysis based on TCGA-ESCC data indicating the close relationship between and the PI3K/AKT pathway. Besides, Zhu et al. (33) confirmed that the phosphorylation level of GSK3β (Ser9) in the PI3K/AKT pathway is closely associated with the malignant phenotype and poor prognosis of ESCC. Multiple studies (34, 35) have demonstrated that this pathway plays a central role in regulating radioresistance in ESCC. Yin et al. (36) found that radiotherapy activates the PI3K/AKT pathway in ESCC cells, promoting the accumulation of myeloid-derived suppressor cells and forming a metastasis-promoting tumor microenvironment. They proposed that combining PI3K/AKT pathway inhibitors with radiotherapy could improve the prognosis of ESCC patients, highlighting the close relationship between the PI3K/AKT signaling pathway and radiotherapy efficacy in ESCC. AKT and GSK3β are core regulatory molecules of the PI3K/AKT/mTOR signaling pathway, playing important roles in tumor development and progression (37). Jayaprakash Kotha et al. (38) demonstrated in CHO-K1 cells that CD9 promotes phosphorylation of AKT (Ser473), a key substrate of PI3K. Based on the above evidence, this study clarified that CD9 works by activating the AKT/GSK3β signaling pathway. In CD9 overexpressing cells, the levels of p-AKT (Ser473) and p-GSK3β (Ser9) were significantly increased, while knocking down CD9 resulted in downregulation of both expressions, indicating that CD9 can act as an upstream regulator of the AKT pathway, thereby regulating the malignant behavior and radiosensitivity of ESCCs.

At the clinical level, although CD9 did not show significant prognostic value in the entire TCGA cohort, subgroup analysis showed that patients with low CD9 expression exhibited better survival trends among those receiving radiotherapy, suggesting its potential as a biomarker for radiation therapy response. Furthermore, the immunohistochemical results of our center’s samples also confirmed the correlation between low CD9 expression and longer survival. ROC analysis further confirms that CD9 has high accuracy in predicting 3-year and 5-year survival, suggesting its potential prognostic biomarker value in the radiotherapy population.

## 5 Conclusions

Conclusively, this study reveals the mechanism by which CD9 promotes ESCC progression and radiation resistance through the AKT/GSK3β pathway, emphasizing its clinical value as a potential radiosensitivity predictor and therapeutic target. In the future, the potential for targeted intervention in combination radiotherapy strategies can be further explored.

## Data Availability

The datasets presented in this study can be found in online repositories. The names of the repository/repositories and accession number(s) can be found below: https://ualcan.path.uab.edu/index.html; https://www.xiantaozi.com/.
