# Supplementary material for: CD9 regulates proliferation, invasion, migration and radioresistance of esophageal squamous cell carcinoma by activating AKT/GSK3β signaling pathway
Source: Front Oncol. 2025 Aug 8;15:1625120. doi: 10.3389/fonc.2025.1625120 (PMC12370492; doi:10.3389/fonc.2025.1625120)
Supplement: Supplementary file 1 [file Table1.docx]

Supplementary Material

# S Table 1 The specific primers

CD9

Forward primer: 5 '- CCTGCTGTTCGGATTTAACTTCA-3';

Reverse primer:5 '- TGGTTGAGAGTGAATCGGA-3'.

GAPDH

Forward primer: 5 '- CATGAAGTATGACAACAGCCT-3';

Reverse primer: 5 '- AGTCTTCCAGATACCAAAGT-3'.
